# Long-term outcomes of radiofrequency ablation vs. partial nephrectomy for cT1 renal cancer: A meta-analysis and systematic review

**DOI:** 10.3389/fsurg.2022.1012897

**Published:** 2023-01-06

**Authors:** Linjin Li, Jianlong Zhu, Huan Shao, Laijian Huang, Xiaoting Wang, Wenshuo Bao, Tao Sheng, Dake Chen, Yanmei He, Baolin Song

**Affiliations:** ^1^ Department of Urology, the Third Clinical Institute Affiliated to Wenzhou Medical University, Wenzhou People’s Hospital, Wenzhou, China; ^2^ Department of Urology, Jiaxing Hospital of Traditional Chinese Medicine, Jiaxing, China

**Keywords:** radiofrequency ablation, partial nephrectomy, recurrence, survival, renal cancer

## Abstract

**Background:**

Partial nephrectomy (PN) is one of the most preferred nephron-sparing treatments for clinical T1 (cT1) renal cancer, while radiofrequency ablation (RFA) is usually used for patients who are poor surgical candidates. The long-term oncologic outcome of RFA vs. PN for cT1 renal cancer remains undetermined. This meta-analysis aims to compare the treatment efficacy and safety of RFA and PN for patients with cT1 renal cancer with long-term follow-up of at least 5 years.

**Method:**

This meta-analysis was performed following the PRISMA reporting guidelines. Literature studies that had data on the comparison of the efficacy or safety of RFA vs. PN in treating cT1 renal cancer were searched in databases including PubMed, Embase, Web of Science, and the Cochrane Library from 1 January2000 to 1 May 2022. Only long-term studies with a median or mean follow-up of at least 5 years were included. The following measures of effect were pooled: odds ratio (OR) for recurrence and major complications; hazard ratio (HR) for progression-free survival (PFS), cancer-specific survival (CSS), and overall survival (OS). Additional analyses, including sensitivity analysis, subgroup analysis, and publication bias analysis, were also performed.

**Results:**

A total of seven studies with 1,635 patients were finally included. The treatment efficacy of RFA was not different with PN in terms of cancer recurrence (OR = 1.22, 95% CI, 0.45–3.28), PFS (HR = 1.26, 95% CI, 0.75–2.11), and CSS (HR = 1.27, 95% CI, 0.41–3.95) as well as major complications (OR = 1.31, 95% CI, 0.55–3.14) (*P* > 0.05 for all). RFA was a potential significant risk factor for OS (HR = 1.76, 95% CI, 1.32–2.34, *P* < 0.001). No significant heterogeneity and publication bias were observed.

**Conclusion:**

This is the first meta-analysis that focuses on the long-term oncological outcomes of cT1 renal cancer, and the results suggest that RFA has comparable therapeutic efficacy with PN. RFA is a nephron-sparing technique with favorable oncologic efficacy and safety and a good treatment alternative for cT1 renal cancer.

## Introduction

For patients with localized cT1 renal cancer warranting curative therapy, nephron-sparing treatments are recommended by most guidelines (1–4). Particularly, partial nephrectomy (PN) has become the preferred therapeutic modality for small renal cancer because quite a few clinical observations reported similar oncologic outcomes to radical nephrectomy (5–7). On the other hand, radiofrequency ablation (RFA), a minimally invasive thermal ablation technique with curative potential for solid tumor, was once considered an alternative therapy predominately for patients not amenable to nephrectomy (8, 9).

With the clinical promotion of RFA application and increased number of studies, several meta-analysis further compared RFA and PN in treating renal cancer. A meta-analysis by Pan et al*.* included 16 studies and found that the local tumor recurrence rate in RFA group was higher than that in PN group [odds ratio (OR) = 1.81]. However, the distant metastasis rate was not statistically different between the two groups (OR = 1.63) (10). Yang et al. analyzed the outcome of radiofrequency ablation over partial nephrectomy for renal mass smaller than 4 cm and identified eight eligible studies for analyses from May 2007 to May 2015 (11). They observed no statistical differences between the two groups in 5-year disease-free survival [hazard ratio (HR) = 1.29, 95% CI, 0.71–2.32, *P* = 0.4], local recurrence rate (OR = 0.99, 95% CI, 0.38–2.58, *P* = 0.98), and surgical complications [relative risk (RR) = 0.82, 95% CI, 0.37, 1.80; *P* = 0.62] between RFA and PN. Overall, the oncologic efficacy of RFA vs. PN has been controversial and undetermined.

Previously, the long-term results comparing partial nephrectomy and radiofrequency ablation were very limited. Olweny et al*.* first reported the oncologic outcomes at a minimum of 5 years of follow-up and found that RFA yielded comparable 5-year overall survival (OS), cancer-specific survival (CSS), overall disease-free survival, local recurrence-free survival, and metastasis-free survival to PN in 74 patients (12). After that, the studies from China by Chang et al*.* also reported that RFA had comparable 5-year oncologic outcomes but better preservation of renal function than PN in clinical T1a renal cancer (13) as well as in T1b renal cancer (14). Ji et al*.* also reported 5-year overall, cancer-specific, and disease-free survival rates of 93.3% vs. 94.6%, 98.0% vs. 98.5%, and 97.1% vs. 97.3%, for RFA and PN, respectively (all *P*-value>0.05) (15). Notably, despite the nonsignificant difference in these statistics, there seem to be a trend of a lower oncologic efficacy for RFA. Therefore, the question of whether RFA and PN have similar efficacy for clinical T1 renal cancer remains unsettled. Now, with the increased data from long-term studies in recent years, we performed this meta-analysis and systematic review to further update our knowledge of the long-term outcomes of RFA and partial nephrectomy for cT1 renal cancer.

## Materials and methods

### Literature search

The meta-analysis and systematic review were conducted and reported following the PRISMA guidelines (16, 17). We searched all literature focusing on the comparison of RFA vs. PN in patients with renal cancer with long-term follow-up of at least 5 years in the following databases from 1 Jan 2000 to 1 May 2022: PubMed, Embase, Web of Science, and the Cochrane Library. The following key words were used for the literature search:

- for searching literature focusing on renal cancer: renal, kidney, rcc, nephritic;
- for searching literature focusing on RFA: ablation, RFA, or radiofrequency;
- for searching literature focusing on PN: nephrectomy or surgical or surgery or resection.

In addition, an additional literature search was performed *via* checking the citation lists of the literature identified and recent meta-analysis reviews. Literature was managed by the software Endnotes (version X7). The protocol of this meta-analysis has been registered in the International Prospective Register of Systematic Reviews (PROSPERO, registration ID: CRD42022329446).

### Literature screening

There were two authors who independently reviewed the literature and assessed its eligibility for inclusion. If there was dissonance with the result, further discussion with the third author was conducted. The human-based studies were considered suitable for inclusion according to the PICOS guideline:

- P (Population): patients with clinical T1 renal cancer (either T1a or T1b);
- I (Intervention): patients were treated by RFA;
- C (Comparison): patients were treated by PN;
- O (Outcome): at least one of the following main outcomes should be reported: rate of recurrence, progression (recurrence, metastases, or progression-free survival (PFS), CSS, and OS. Secondary outcome is the rate of major complication;
- S (Study design): a long-term comparative study with median/mean follow-up time longer than 5 years in both the RFA and PN groups.

The following literatures were excluded during the screening of title, abstract and full text:

(1) duplicate literatures;
(2) non-English literatures;
(3) several types of literature that usually do not contain original data: review, meta-analysis, guideline, letter, comment, editorial, reply, and protocol;
(4) case report;
(5) nonrelevant topic; and
(6) no available data were found in the full text review.

### Data extraction

Two authors independently extracted raw data from each study. The third author was responsible for checking the data extracted by the two authors and resolving divergences *via* discussion and literature review. The following raw data from included studies were extracted: study location, stage of renal cancer, ablation approach (percutaneous or laparoscopic), ablation navigation (computed tomography or ultrasound), surgical approach (open or laparoscopic), study sample size, number of the surgery group, number of the ablation group, average age of the entire population, follow-up duration of the surgery group, follow-up duration of the ablation group, and R.E.N.A.L. nephrometry score (18) of both groups. Furthermore, the following data were collected for further data synthesis: the incidence of recurrence in the whole follow-up period; the HR value and 95% CI of PFS, CSS, and OS; and the incidence of major complications. If the HR and 95% CI were unavailable but the Kaplan–Meier (K–M) curve were provided for PFS, CSS, or OS, then the statistics of time-to-event were extracted from the Kaplan–Meier curve by using the software Engauge, and the data were further used to calculate the HR and 95% CI *via* the method provided by Tierney et al. (19). All extracted data are collected in an Excel file, which can be found in the Supplementary Material.

### Definitions

#### Recurrence

Local recurrence was defined as a new focal enhancement in the ablation bed or enlargement of the ablation defect on follow-up imaging for RFA and a new mass at or near the PN site for PN. Metastatic recurrence was defined as extrarenal disseminated disease, with or without pathologic confirmation. Tumor recurrence included local recurrence and metastatic recurrence.

#### Progression-free survival

PFS was defined as the period from the date of treatment start or the baseline assessment until objective disease progression, subjective disease deterioration, or death, whichever occurred first.

#### Cancer-specific survival

CSS was defined as the duration from the time of treatment start or the baseline assessment to the date of renal cancer-related death or the end of follow-up.

#### Overall survival

OS was defined as the duration from the time of treatment start or the baseline assessment to the date of death or censor of follow-up.

#### Major complication

Postoperative complications were categorized according to the Common Terminology Criteria Adverse Events (CTCAE) version 5.0:

(1) Grade 1: Mild adverse events (AEs); asymptomatic or mild symptoms; requiring no treatment;
(2) Grade 2: Moderate AEs; requiring less treatment; local or noninvasive treatment;
(3) Grade 3: Severe AEs but not immediately life-threatening; hospitalization or prolong of hospitalization;
(4) Grade 4: Life-threatening; requiring emergency treatment;
(5) Grade 5: Death due to AEs.

Major complications were considered CTCAE grade ≥3.

### Study quality and risk of bias assessment

Based on the recommendations of Cochrane Collaborations, two independent authors evaluated the quality of the included studies using the ROBINS-I risk of bias assessment tool (20) which consists of seven domains, namely, bias due to confounding, bias in the selection of participants into the study, bias in the classification of interventions, bias due to deviations from intended interventions, bias due to missing data, bias in measurement of the outcome, and bias in the selection of the reported result (21). The dissonance of the results was resolved in a similar way as described in the Literature search section. The risk of overall bias was assessed according to the summary of the above items.

### Effect measures and synthesis methods

In the synthesis and presentation of results, the following effect measures were obtained using the *metan* module of the STATA software, version 15 (Stata Corporation, College Station, TX, United States): OR and 95% CI for recurrence and major complications, HR and 95% CI for PFS, CSS, and OS. The studies were eligible for each synthesis when the relative raw data were available following the random effects model. For missing values such as the HR for PFS, CSS, and OS, the statistics were extracted from the Kaplan–Meier curves as described above in the Data extraction section. The forest plots were used to visually display the results of individual studies and syntheses. Subgroup analysis was performed to explore possible causes of heterogeneity among the study results. The studies were divided into subgroups according to the following factors: study location (United States, China, and Korea), stage of renal cancer (T1a, T1b, and T1a/T1b), ablation approach (percutaneous and laparoscopic), ablation navigation (CT and ultrasound), surgical approach (open and laparoscopic), average age (≤60 and >60 years), and R.E.N.A.L. nephrometry score (available and not reported). Sensitivity analysis was conducted by omitting one literature at each analysis to evaluate the robustness of the synthesized results using the *metaninf* module of the STATA software.

### Reporting bias assessment

To assess the risk of bias due to missing results in a synthesis, the *metabias* module of STATA was used to perform *Egger'*s test. The *P*-value of *Egger'*s test <0.05 was considered significant publication bias. The funnel plot for identifying the underreported articles was also performed by using the *metafunnel* module of STATA to visually display the results of the reporting bias assessment.

## Results

### Study selection and characteristics of included studies

**Figure 1:**
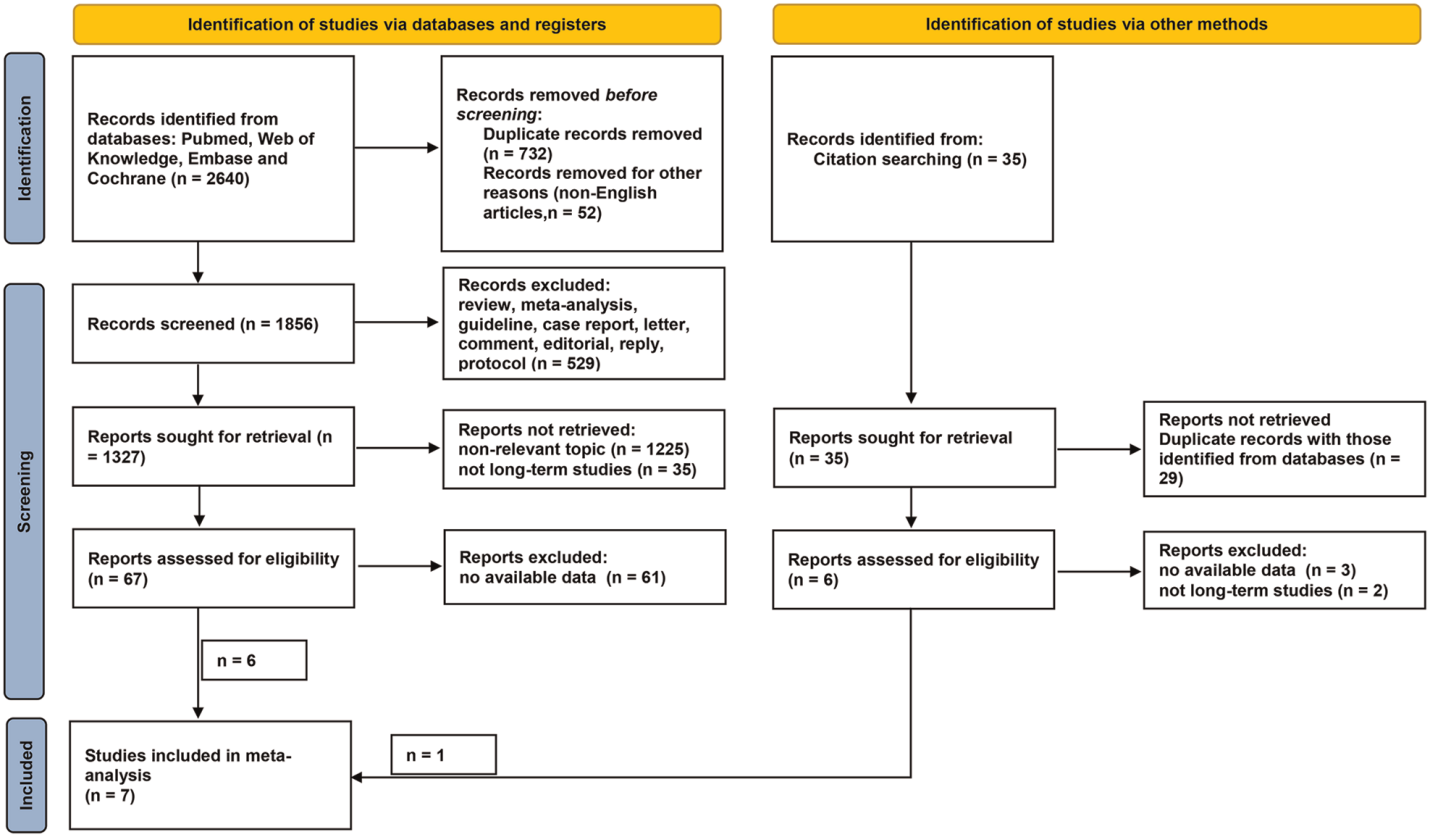
Flow diagram of literature selection. Records were identified *via* databases (*n* = 2,640) and other methods (citation searching, *n* = 35). After screening, a total of six and one records were considered eligible for inclusion from databases and other methods, respectively. Finally, seven studies were included in the present meta-analysis.

As shown in Figure 1, a total of 2,640 and 35 studies were initially identified from database searching and citation searching, respectively. After screening by reviewing the title, abstract, and full text, seven studies were finally included in the meta-analysis (12–15, 22–24). As listed in Table 1, 1,635 patients with renal cancer (548 in the RFA group and 1,413 in the PN group) were included. Four studies were conducted in China, two in the United States, and one in Korea. Both T1a and T1b renal cancers were studied. RFA was performed either percutaneously or laparoscopically. Ultrasound was the most commonly used navigation technique for RFA (*n* = 6 out of 7) while PN was performed either open or laparoscopically. The median/mean follow-up duration ranged from 60 to 90 months for the RFA group and 66 to 113 months for the PN group. The characteristics of the PN and RFA groups were compared and listed in Table 2. The R.E.N.A.L. nephrometry scores of both groups were reported in three studies. In two studies, the nephrometry scores were similar between two groups (mean/median score = 8 in both groups). In another study, the nephrometry scores were not different (8.5 in the PN group and 7.8 in the RFA group, *P* = 0.698). In most of the studies (*n* = 6 out of 7), the patients in RFA group were significantly older than PN group. Similarly, in five of the seven studies, the patients in RFA group had higher ASA scores. We have also collected the indication for the choice of treatment approach (PN and RFA) in these studies, and it turned out that RFA was commonly recommended in patients with significant comorbidities, a solitary kidney, or tumors in unresectable locations.

**Table 1:** Characteristics of included studies.

Study	Study location	Stage of RCC	RFA approach	RFA navigation	PN approach	Study sample size	No. of PN group	No. of ablation group	Average age	Follow-up of PN group	Follow-up of RFA group
Olweny (2012)	United States	T1a	P/L	CT	O/L	74	37	37	<60	66	61
Chang (2015)^a^	China	T1b	P/L	US	O/L	56	29	27	>60	71	73
Chang (2015)^b^	China	T1a	P/L	US	O/L	90	45	45	<60	69	67
Ji (2016)	China	T1a	L	US	L	179	74	105	>60	82	78
Liu (2017)	China	T1a/T1b	P	US	O/L	213	120	93	>60	80	77
Park (2019)	Korea	T1a	L	US	O	115	53	62	<60	68	60
Andrews (2019)	United States	T1a	P	US/CT	O	908	835	73	>60	113	90

RCC, renal cell carcinoma; RFA, radiofrequency ablation; PN, partial nephrectomy; P/L: percutaneous or laparoscopic; L, laparoscopic; P, percutaneous; CT, computed tomography; US, ultrasound; O/L, open or laparoscopic; O, open.

^a^ Chang et al. in 2015 investigated the T1b stage RCC.

^b^ Chang et al. in 2015 investigated the T1a stage RCC.

**Table 2:** Comparison of PN and RFA groups.

	R.E.N.A.L. nephrometry score (mean/median + range)		Mean/median age (year)		Mean/median ASA score		Choice of treatment approach	
Study	PN group	RFA group	PN group	RFA group	PN group	RFA group	Indication for PN	Indication for RFA
Olweny (2012)	N/A	N/A	54.8	63.8	1.9	2.3	Unspecified	Unspecified
Chang (2015)^a^	7.8 (5–11)	8.5 (6–11)	56.9	64	1.5	2.1	Unspecified	Unspecified
Chang (2015)^b^	8 (5–10)	8 (6–10)	52.8	52.9	1.7	1.7	Unspecified	Patients with significant comorbidities, a solitary kidney, or tumors in unresectable locations; patients unwilling to take the risk of PN
Ji (2016)	N/A	N/A	57.3	64.2	1.7	2.3	Unspecified	Older and comorbid patients; the presence of solitary kidney
Liu (2017)	8 (5–11)	8 (5–11)	58.5	68	1	2	Unspecified	Patients with smaller tumors (<4 cm) and peripheral tumors
Park (2019)	N/A	N/A	53	58	1.6	1.8	Unspecified	Unspecified
Andrews (2019)	N/A	N/A	62	72	N/A	N/A	Eligibility for PN was first determined by the urologist's discretion	Patients further interested in percutaneous ablation or deemed unfit for PN

PN, partial nephrectomy; RFA, radiofrequency ablation; R.E.N.A.L., radius, exophytic/endophytic, nearness of tumor to collecting system, anterior/posterior, hilar tumor touching main renal artery or vein and location relative to polar lines; ASA, American Society of Anesthesiologists score; N/A, not available.

^a^ Chang et al. in 2015 investigated the T1b stage RCC.

^b^ Chang et al. in 2015 investigated the T1a stage RCC.

### Risk of bias in studies

**Figure 2:**
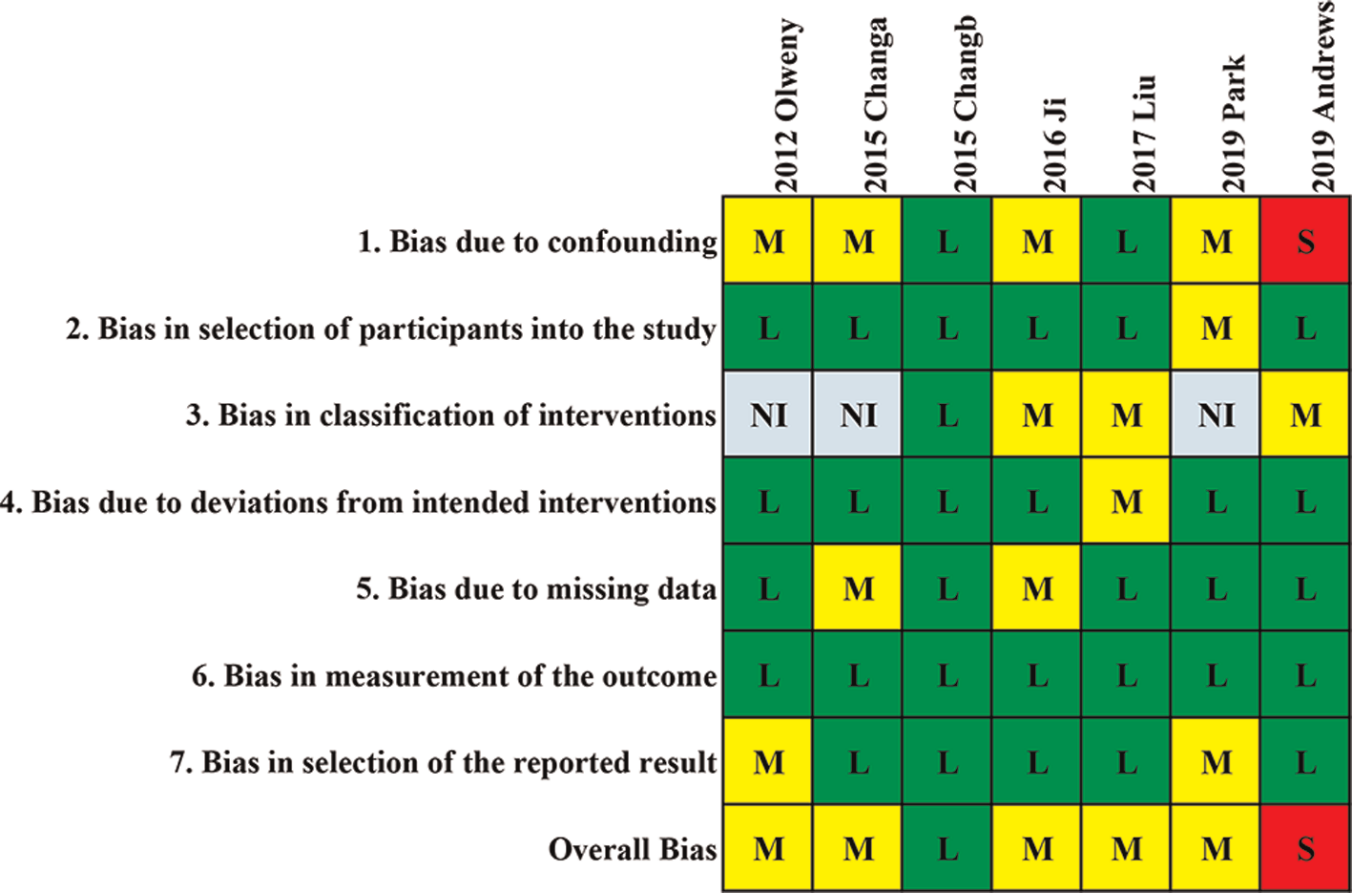
Quality assessment of included studies. Quality assessment of the involved studies with a ROBINS-I tool for non-RCT studies. L, low; M, moderate; S, serious; NI, no information.

Figure 2 shows the results of the assessments of study risk by using the ROBINS-I tool for non-randomized controlled trial (RCT) studies. Overall, one study was of high quality with low risk of bias, five studies were of moderate quality, and the other one study was identified as having low quality. The most common confounding bias risk was due to the different ages in the RFA and PN groups. The confounding bias risk in the study by Andrews et al. was considered serious due to distinct baseline confounding factors including age, serum creatinine, histology, and size of tumor. The study by Chang et al. was evaluated as high quality because it was designed based on a propensity score-matched cohort, which reduced the risk of confounding factors.

### Results of individual studies and syntheses, sensitivity analyses, and reporting biases

#### Recurrence

**Figure 3:**
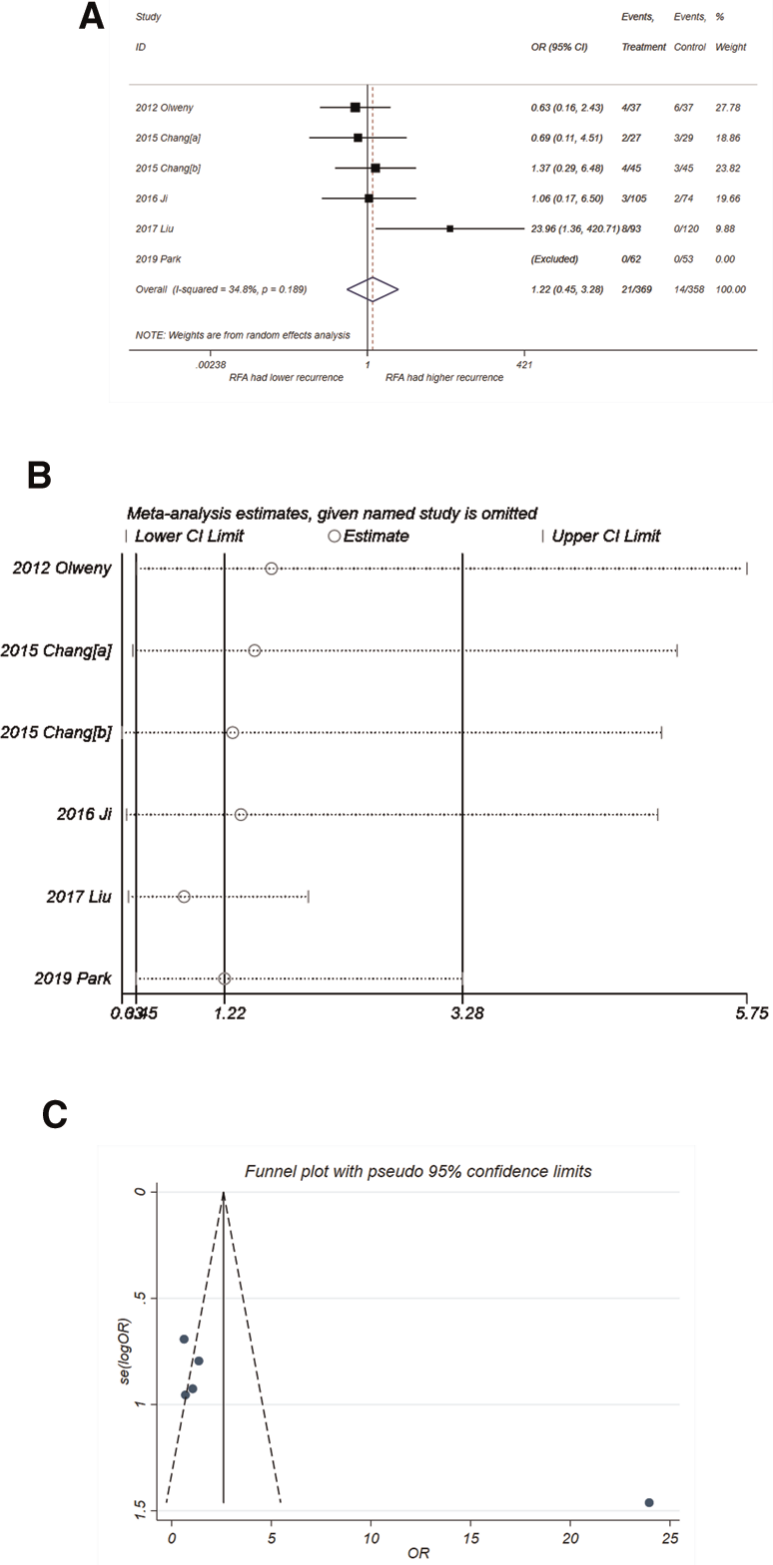
Comparison of recurrence incidence of RFA vs. PN. (**A**) The forest plot shows the OR (odds ratio) of recurrence incidence of RFA vs. PN. OR > 1 indicates that RFA has higher probability of recurrence. (**B**) Sensitivity analysis was performed by omitting one study at each analysis. The result of each analysis is also presented as the forest plot. (**C**) The funnel plot was used to detect publication bias. RFA, radiofrequency ablation; PN, partial nephrectomy; OR, odds ratio.

As shown in Figure 3A, RFA had a higher recurrence probability than PN but without statistical difference (OR = 1.22, 95% CI, 0.45–3.28, *P* = 0.691) with minor heterogeneity (*I*^2^* *= 34.8%, *P* = 0.189). The sensitivity analysis (Figure 3B) showed that the study by Liu et al. (22) had a distinct impact on the pooled result while the other studies did not. After omitting the study by Liu et al*.*, the pooled OR was 0.87 (95% CI, 0.39–1.95), which favors the treatment of RFA but still without significant difference. The funnel plot (Figure 3C) also suggested that that the study by Liu et al*.* was a potential source of publication bias, but this bias did not reach statistical significance (*P-*value of *Egger*'s test = 0.063).

#### Progression-free survival

**Figure 4:**
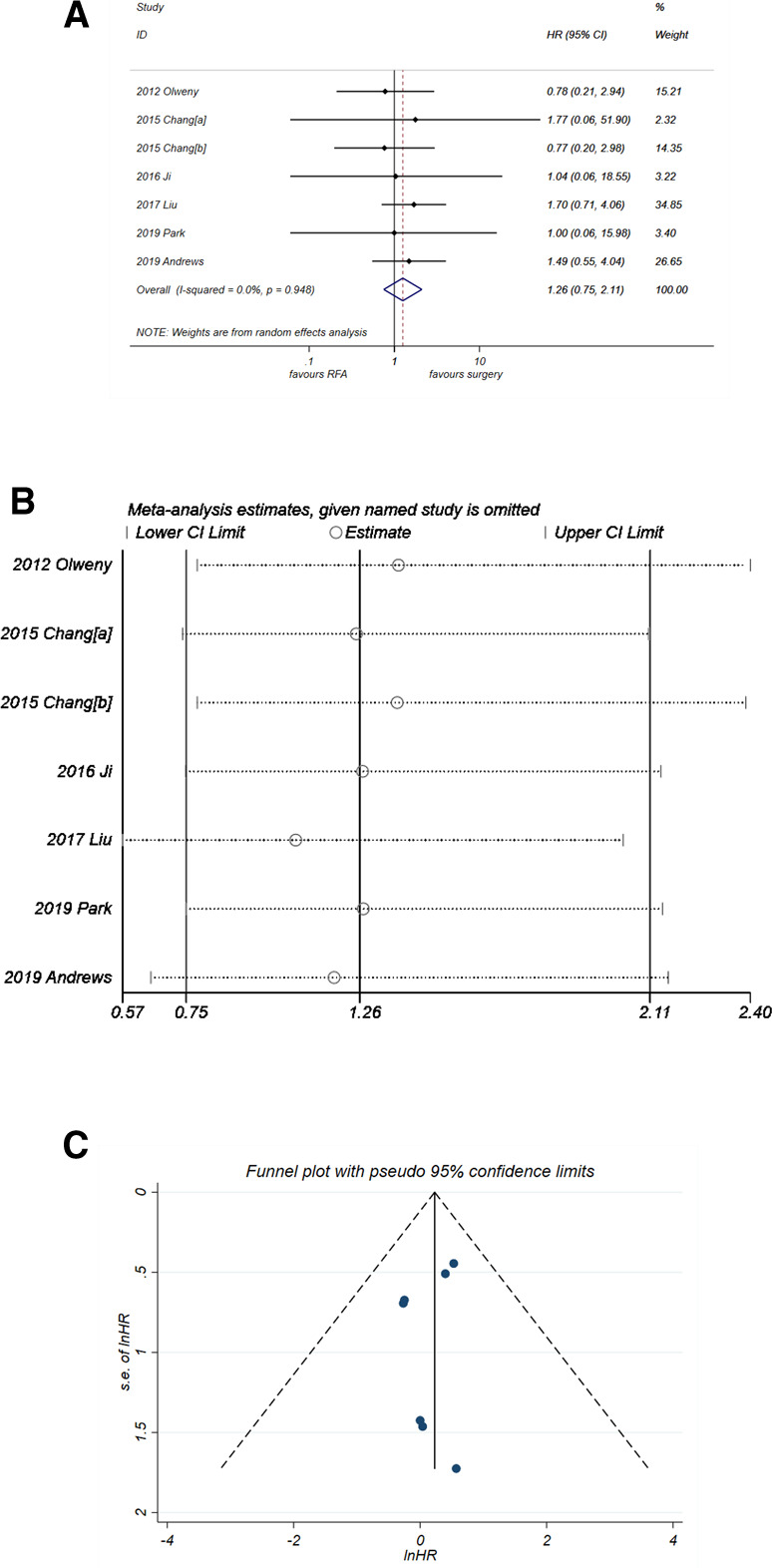
Comparison of PFS of RFA vs. PN. (**A**) The forest plot shows the HR of PFS of RFA vs. PN. HR > 1 indicates that RFA has higher risk for PFS. (**B**) Sensitivity analysis was performed by omitting one study at each analysis. The result of each analysis is also presented as the forest plot. (**C**) The funnel plot was used to detect publication bias. PFS, progression-free survival; RFA, radiofrequency ablation; PN, partial nephrectomy; HR, hazard ratio.

RFA might be a potential risk factor for PFS (Figure 4A), with a pooled HR of 1.26 (95% CI, 0.75–2.11) but without significance (*P* = 0.382). There was no heterogeneity observed (*I*^2^* *= 0%, *P* = 0. 948). The sensitivity analysis (Figure 4B) suggested that the result of pooled PFS was relatively stable. The funnel plot also showed good symmetry, and the *P*-value of *Egger'*s test was 0.443. Thus, no publication bias was considered.

#### Cancer-specific survival

**Figure 5:**
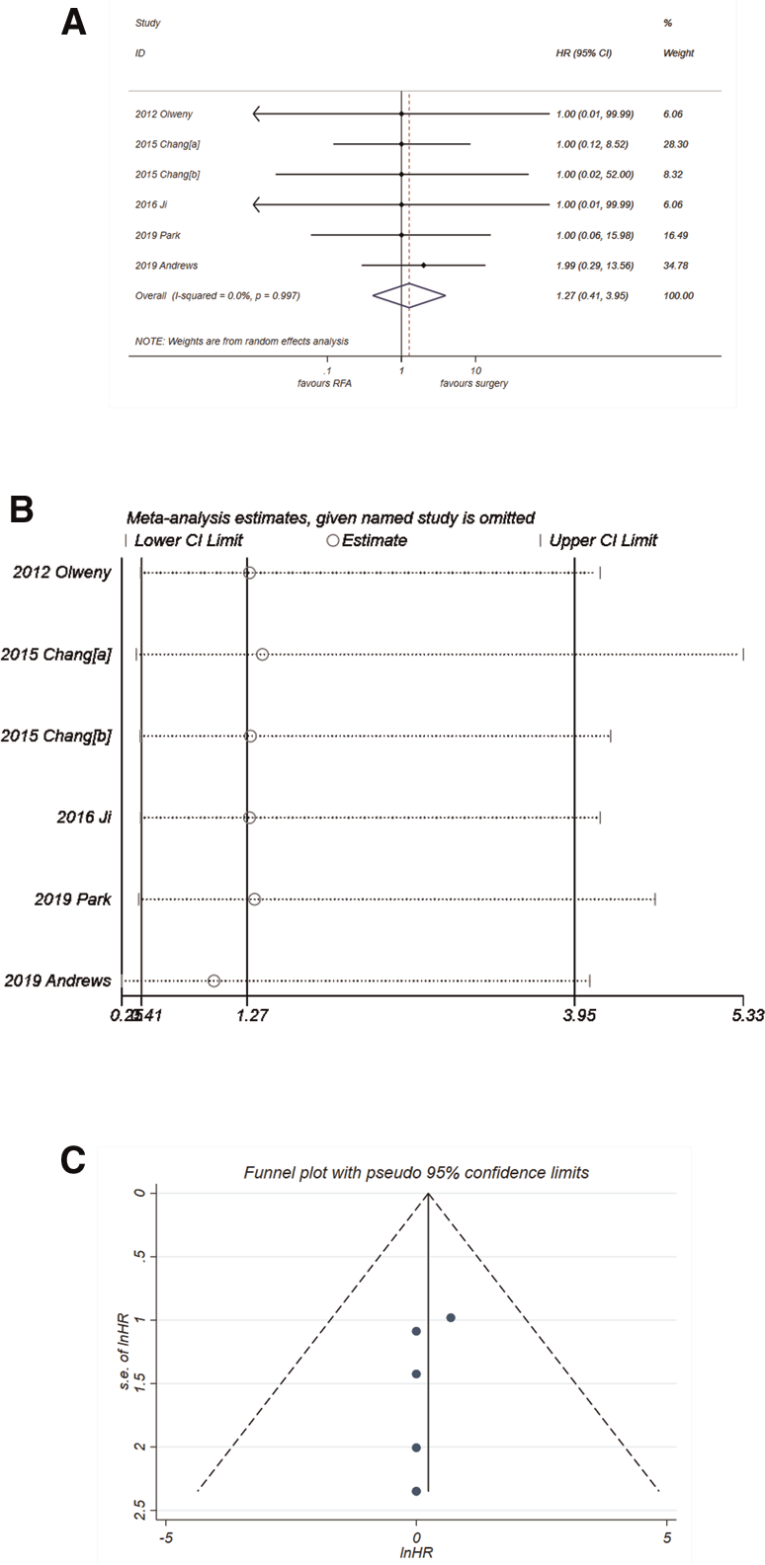
Comparison of CSS of RFA vs. PN. (**A**) The forest plot shows the HR of CSS of RFA vs. PN. HR > 1 indicates that RFA has higher risk for CSS. (**B**) Sensitivity analysis was performed by omitting one study at each analysis. The result of each analysis is also presented as the forest plot. (**C**) The funnel plot was used to detect publication bias. CSS, cancer-specific survival; HR, hazard ratio; PN, partial nephrectomy; RFA, radiofrequency ablation.

The results of CSS were consistent with those of PFS. The pooled HR (Figure 5A) was 1.27 (95% CI, 0.41–3.95) with *P* = 0.679. No heterogeneity was observed (*I*^2^* *= 0%, *P* = 0. 997). The forest plot in Figure 5B showed good robustness of the synthesized HR of CSS. Similarly, no publication bias was found in the funnel plot in Figure 5C (*P*-value of *Egger's* test was 0.262).

#### Overall Survival

**Figure 6:**
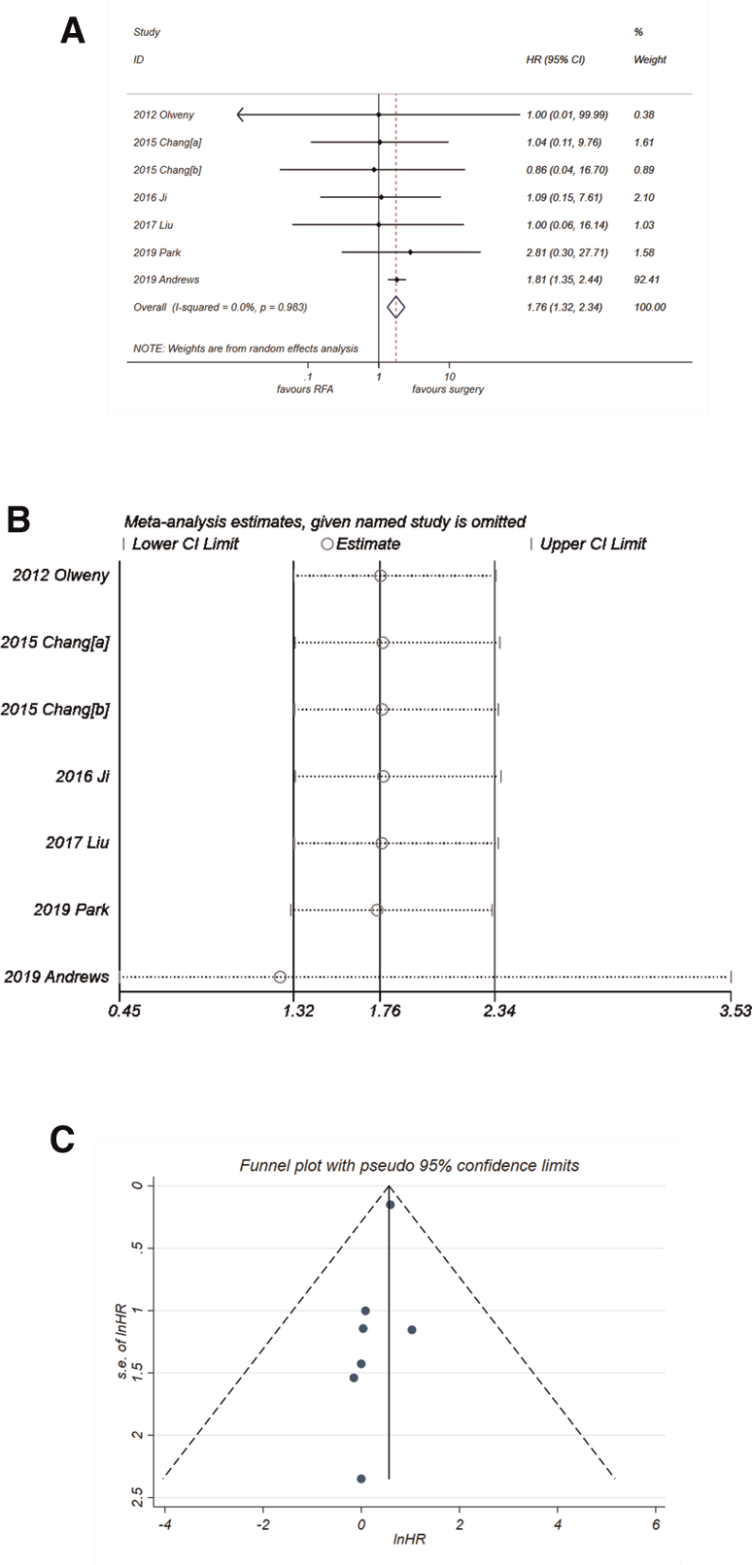
Comparison of OS of RFA vs. PN. (**A**) The forest plot shows the HR of OS of RFA vs. PN. HR > 1 indicates that RFA has higher risk for OS. (**B**) Sensitivity analysis was performed by omitting one study at each analysis. The result of each analysis is also presented as the forest plot. (**C**) The funnel plot was used to detect publication bias. HR, hazard ratio; OS, overall survival; PN, partial nephrectomy; RFA, radiofrequency ablation.

Unlike PFS and CSS, analysis of OS (Figure 6A) suggested that RFA was a significant risk factor with synthesized HR = 1.76 (95% CI, 1.32–2.34, *P* < 0.001). No heterogeneity was observed (*I*^2^* *= 0%, *P* = 0. 983). The sensitivity analysis (Figure 6B) showed that the robustness of the synthesized HR of OS was fine. A good degree of symmetry was noticed *via* the funnel plot (Figure 6C) with a *P*-value of *Egger's* test = 0.099, indicating no significant publication bias.

#### Major complication

**Figure 7:**
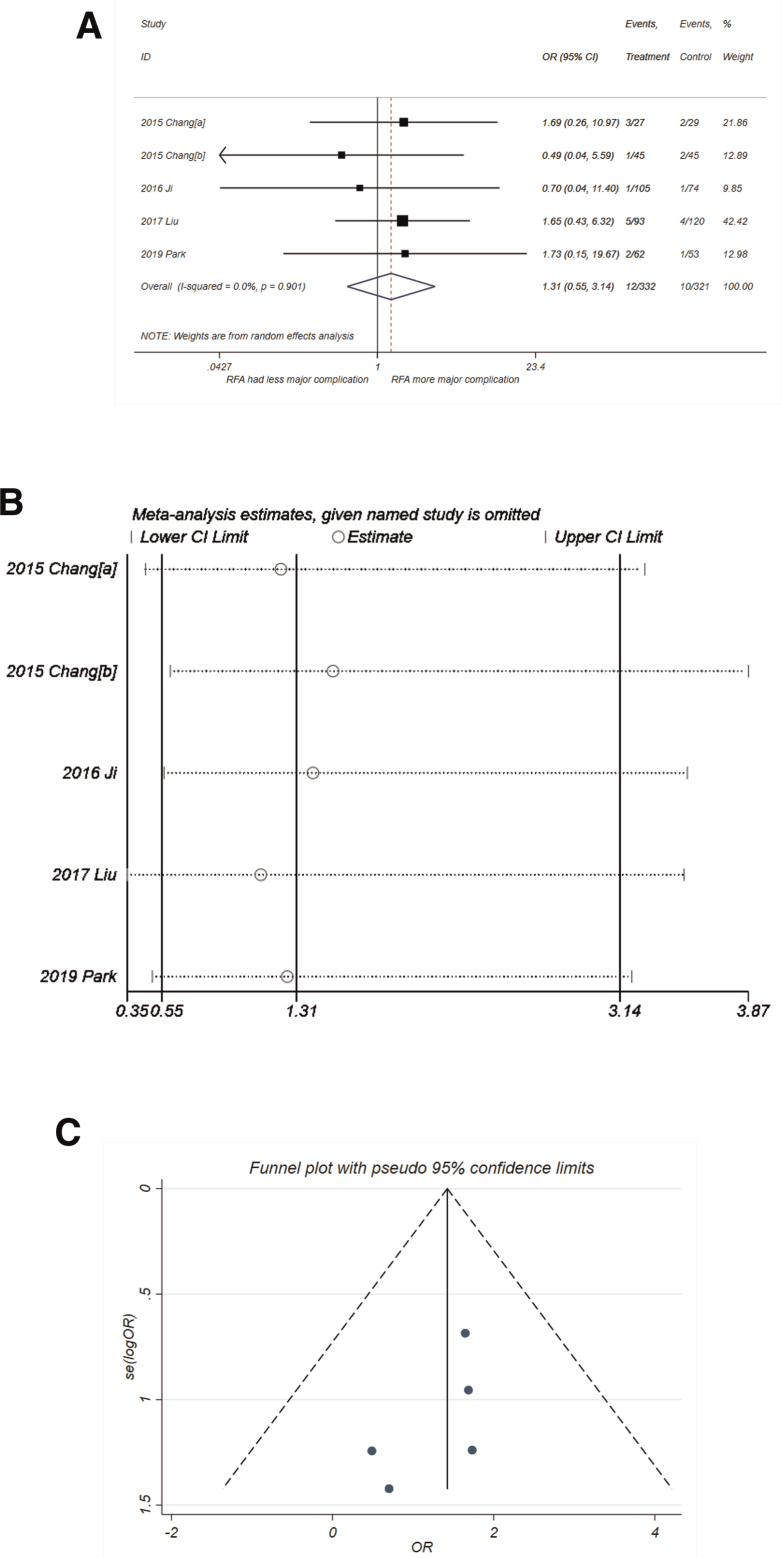
Comparison of major complication of RFA vs. PN. (**A**) The forest plot shows the OR of major complication of RFA vs. PN. OR > 1 indicates that RFA has higher probability of major complication. (**B**) Sensitivity analysis was performed by omitting one study at each analysis. The result of each analysis is also presented as the forest plot. (**C**) The funnel plot was used to detect publication bias. PN, partial nephrectomy; RFA, radiofrequency ablation; OR, odds ratio.

No significant difference in the incidence of major complication was observed for RFA and PN (Figure 7A) with OR = 1.31 (95% CI,0.55–3.14, *P* = 0.545). No heterogeneity was observed (*I*^2^* *= 0%, *P* = 0. 901). The sensitivity analysis (Figure 7B) showed good robustness of the result. No publication bias was observed in the funnel plot in Figure 7C (*P*-value of *Egger'*s test was 0.228).

### Subgroup analysis

To investigate the potential source of heterogeneity for the result of recurrence, further subgroup analysis was performed. Because no heterogeneity was observed for PFS, CSS, OS, and major complication, subgroup analysis was performed only for recurrence. As shown in Table 3, because of the small number of included studies, the analysis was not valid in most of the subgroups. However, the values of *I*^2^ in the subgroups of average age ≤60 and >60 years were 0.0% and 61.1%, respectively, indicating that the three studies with average population age >60 years might be the major source of heterogeneity for recurrence.

**Table 3:** Subgroup analysis for recurrence.

Subgroup	No. of studies	OR	Lower 95% CI	Upper 95% CI	*I* ^2^	*P* for *I*^2^
Study location						
United States	1	0.626	0.161	2.432	—	—
China	4	1.629	0.461	5.748	40.7%	0.167
Korea	1	—	—	—	—	—
Stage of RCC						
T1a	4	0.918	0.376	2.237	0%	0.749
T1b	1	0.693	0.107	4.505	—	—
T1a/T1b	1	23.959	1.364	420.711	—	—
Ablation approach						
Percutaneous or laparoscopic	3	0.831	0.338	2.038	0%	0.743
Laparoscopic	2	1.059	0.173	6.499	—	—
Percutaneous	1	23.959	1.364	420.711	—	—
Ablation navigation						
CT	1	0.626	0.161	2.432	—	—
US	5	1.629	0.461	5.748	40.7%	0.167
Surgical approach						
Open or laparoscopic	4	1.363	0.374	4.976	51.6%	0.102
Laparoscopic	1	1.059	0.173	6.499	—	—
Open	1	—	—	—	—	—
Average age						
≤60	3	0.877	0.315	2.439	0%	0.459
>60	3	2.006	0.279	14.412	61.1%	0.077
R.E.N.A.L. nephrometry score						
Available	3	2.118	0.341	13.162	59%	0.087
Not reported	4	0.756	0.255	2.241	0	0.65

OR, odds ratio; CI, confidence interval; RCC, renal cell carcinoma; CT, computed tomography; US, ultrasound. R.E.N.A.L., radius, exophytic/endophytic, nearness of tumor to collecting system, anterior/posterior, hilar tumor touching main renal artery or vein and location relative to polar lines.

## Discussion

In the present meta-analysis, RFA showed lower OS but similar recurrence, PFS, CSS, and major complications as compared with PN during the long-term follow-up over 5 years. This is currently the first meta-analysis focusing on the long-term outcomes of RFA and PN for renal cancer. It demonstrates the therapeutic efficacy as well as the safety of RFA for patients with renal cancer, especially for those not amenable to surgery.

Several guidelines have already recommended that thermal ablation should be considered in patients with small-size cancers who are poor surgical candidates (25–27). The puncture procedure of percutaneous RFA is similar to a needle biopsy and involves inserting a needle-like probe into the organ (28). Then, radiofrequency waves are produced by the probe and sent into the nearby tissue, which causes the necrosis of surrounding cells (29). Thereby, this relatively new technique has a remarkable advantage over PN, namely, RFA is better at preserving renal function as well as reducing other perioperative and postoperative complications (30, 31). Thereafter, many clinical trials and observations have reported favorable results with RFA when compared with PN. For instance, Bird et al. compared laparoscopic-guided RFA with laparoscopic PN in a retrospective study containing 69 patients and found no evidence of tumor recurrence in the follow-up period (32). In a large cohort study by Thompson et al., they reported that local recurrence-free survival and metastases-free survival were not significantly different between percutaneous RFA and PN (33). One of the shortcomings of most studies is the limited number of events (including local recurrence, distant metastases, death, and cancer-specific death), which is mainly due to the short duration of follow-up (34). It is difficult to yield a statistically significant difference within short-term of follow-up; thus, a long-term study is needed to further determine the oncologic efficacy of RFA and PN. A recent meta-analysis by Rivero et al. (35) has a very alike theme with our meta-analysis. However, only 3 of the 15 studies included in the meta-analysis were with long-term follow-up more than 5 years. The results of the meta-analysis mainly reflect short/mid-term outcome of ablation vs. PN. There are several similar findings between the meta-analysis and ours. For instance, both meta-analyses yield a more favorable overall survival of PN. The results of cancer recurrence in both meta-analyses were similar between PN and ablation (HR= 1.32 and 1.22, *P* = 0.22 and 0.691, respectively). However, the cancer-specific survival was almost similar between RFA and PN in our study (HR = 1.27, *P* = 0.679), but in their meta-analysis, PN showed better efficacy for cancer-specific survival with HR of 3.84 (*P* < 0.05). Because ablation is usually applied to relatively older patients with more underlying diseases, this can lead to more noncancer-related death cases during the long-term follow-up. Therefore in our meta-analysis, the OS result favors PN, but the cancer-specific survival is similar between PN and RFA. The results of CSS might reflect a more objective comparison of RFA vs. PN.

An important interpretation for the results of the present meta-analysis is that although RFA shows an HR of 1.76 (*P* < 0.001), it does not necessarily mean that the efficacy of RFA is worse than PN. First, because the HR of OS in the six studies (Figure 6A) other than Andrews et al*.* (24) is extracted from the K–M curve, they all show large 95% CI range with a small weight in the synthesized data. On the other hand, the study by Andrews et al*.* with HR of 1.81 (95% CI, 1.32–2.34) holds a big weight of 92.4% in the synthesized data, as shown in Figure 6A. Thereby, the synthesized result is mainly affected by the study of Andrews et al*.*, which leads to a final HR of 1.76 (*P* < 0.001). There might be more or less some bias in this figure, which is mainly due to the data extraction method and is hard to eliminate. Similarly, in the analysis of CSS (Figure 5A), the study by Andrews et al*.* holds a weight of 34.8%; this is way smaller than that in the analysis of OS (92.4%, Figure 6A). The final synthesized result of CSS suggests that the efficacy of RFA is not different with PN. Taken together, these results indicate that there might be some factors other than cancer-specific factors inducing a lower OS of RFA, such as age. As depicted in the characteristics of the seven included studies, six of them included an older population of the RFA group. Therefore, there might also be a potential selection bias, which can lead to the inconsistent results of OS and CSS.

Currently, PN is the treatment of choice for cT1 renal cancer, while RFA is considered an alternative therapy for patients with high surgical risk (2). The preference for PN might reflect the relative lack of clinical studies investigating the long-term oncological outcomes of RFA. Nevertheless, in this meta-analysis, with several recently published studies, we show that RFA and PN for cT1 renal cancer have comparable long-term oncological outcomes. There are several strengths of the present work: there is only minor heterogeneity in the analysis of recurrence and zero heterogeneity in other analysis, suggesting that the synthesis of data is more convincing; most of the results in the sensitivity analysis are quite stable, further demonstrating the robustness of the synthesized results; and lastly, the possibility of publication bias is extremely low, as demonstrated by the funnel plots and *Egger'*s tests.

There are also several limitations to this study. The number of included studies is relatively small, which is mainly due to the current clinical practice of renal cancer management. In addition, despite the fact that the choice of approach was usually based on tumor size, location, clinical judgment, and patient preference, it turned out that RFA was commonly recommended in patients with significant comorbidities, solitary kidney, or tumors in unresectable locations. Therefore, RFA was mainly performed in older patients with more preoperative risk factors who were not surgical candidates, which might contribute to the selection bias. A randomized controlled trial could be ideal and is expected to be performed in the future. Despite these limitations, the results in most studies have supported the clinical usefulness of RFA in appropriately selected patients with renal cell carcinoma (RCC).

## Conclusion

This meta-analysis, which focuses on the long-term oncological outcomes of cT1 renal cancer, suggests that RFA has comparable therapeutic efficacy to PN. RFA is a safe, nephron-sparing, and oncologically effective technique for the treatment of cT1 renal cancer and also a potential treatment alternative for the young, healthy population. Nevertheless, a prospective randomized study with large number of patients and long-term follow-up could draw a further conclusion.

## Data Availability

The original contributions presented in the study are included in the article/**Supplementary Material**, further inquiries can be directed to the corresponding author.
